# Travellers with prosthetic limbs, a neglected population. A perspective on what travel health practitioners need to know

**DOI:** 10.1186/s40794-024-00226-z

**Published:** 2024-08-15

**Authors:** Irmgard L. Bauer, Vikranth H. Nagaraja

**Affiliations:** 1 College of Healthcare Sciences, Academy - Tropical Health and Medicine, James Cook University, Townsville, QLD 4811 Australia; 2 Centre for Human Movement and Rehabilitation, School of Health and Society, University of Salford, Salford, M5 4WT UK; 3 Department of Engineering Science, Institute of Biomedical Engineering, Oxford University, Old Road Campus Research Building, Headington, Oxford, Oxfordshire OX3 7DQ UK

**Keywords:** Travel health, Artificial limb, Amputee, Disability, Body image, Airport security, Travel barriers, Travel guidelines.

## Abstract

**Background:**

The benefits of travel for the wellbeing of people of all ages and abilities are well known, though travellers with prostheses have so far been excluded. Limb loss, due to trauma, vascular disease, cancer, or infections requires a prosthesis for cosmesis and functionality. The life-changing event of losing a limb and the considerable psychological adjustment to accept an altered body image influence rehabilitation and self-management as well as the participation in social activities, such as sport and travel. The challenge of travel lies not only in transferring practical impediments encountered at home to another location; familiar coping strategies may require unexpected adjustments. After presenting background information on limb loss and prostheses, the purpose of this paper was to review literature on health advice for travellers with prosthetic limbs.

**Method:**

All major data bases were searched for peer-reviewed literature using a variation of keyword combinations around travel and prosthetics. Relevant journals were searched individually, and selected authors and university departments contacted. No evidence-based results were obtained. The search then moved to grey literature including documents from relevant organisations, professional bodies, government websites, manufacturers, airlines, prosthetic/physiotherapy clinics, sport organisations to approaching amputees, including veterans and athletes, directly.

**Result:**

The list of collated travel advice for people with artificial limbs relates to (1) trip preparation, (2) packing (especially considering the mechanical and/or electrical requirements of the prosthesis), (3) travelling by plane as the most covered mode of travel, and (4) navigating airports and airport security, which may be used by travel health practitioners while awaiting evidence-based guidelines.

**Conclusion:**

This is the first paper on travel with a prosthetic limb in any field, including travel medicine. Therefore, travel health practitioners have no evidence-based guidelines at their disposal required for high-quality care for this neglected population. Preliminary recommendations for clinical practice, advice for required updates in education, and suggestions for urgently needed research are provided to replace current hints and tips with evidence so that travellers with prostheses are no longer ‘out on a limb’.

**Table:** 

**What is known of this topic?** There appears to be no academic literature on travelling with a prosthetic limb in any field, including travel medicine. **What does this paper add?** This paper alerts to the challenges amputees face when travelling and provides background information for suggestions for pertinent travel health advice.
**What further research is needed?** Without current scientific evidence, countless topics invite investigation, including travelling amputees’ perceptions and coping mechanisms with barriers and concerns, practical aspect of travel and transport, feasible tourist activities, stump and prosthesis care, and self-management, to name but a few. Clinicians’ experiences with amputees and advice given are yet unknown.

## Introduction

The benefits of travel for the wellbeing of people of all ages and abilities are well known [1]. The tourism and hospitality industry caters increasingly for travellers with disabilities, either forced by government mandates, by understanding special-needs travellers as a business opportunity, or by responding to public pressure. A growing body of knowledge, especially in tourism academia, relates to the many aspects of travelling with a disability, predominantly sensory or mobility impairment [2, 3], including a focus on transport [4], gender [5] or staff accompanying passengers [6]. However, most literature refers to travellers in wheelchairs, not those with other specific needs, e.g., with prosthetic limbs. This vulnerable group of travellers has been ignored in the travel medicine literature, apart from a fleeting mention at the First International Conference on Travel Medicine (CISTM1) in 1988 relating to travel on freighters [7]. Prosthetic users might be interested solely in travelling for work or personal reasons or to a destination for leisure, adventure, sports, recreation and/or extreme travel. Undoubtedly, travelling with prostheses that would be used in demanding conditions, in resource-constrained and/or remote regions brings its unique challenges, warranting expert advice, proactivity, and careful preparation and planning.

Limb amputations adversely affect individuals’ quality of life (QOL), having significant functional, mobility, psychological, economic, and/or social implications. Further, acquired upper limb (UL) loss is more devastating to an individual than lower limb (LL) loss. Additionally, limb amputation considerably impacts lifestyle and self-perception. Although precise data are difficult to obtain, the Global Burden of Disease Study 2019 estimated a prevalence of approximately 176 million amputees worldwide [8]. Limb loss in the US alone by 2050 is estimated at 3.6 million [9]. While limb loss can be congenital, acquired amputations of ULs and LLs are caused by trauma, peripheral vascular disease (e.g., diabetes), malignancy, infections (meningitis, gangrene, polio), or malformation. In 2017, approximately 58 million people had traumatic amputations with the number of unilateral LL amputations leading unilateral UL, bilateral UL, and bilateral LL cases [10]. Limb loss due to trauma is caused by work or road accidents, gunshots, war and conflict, landmines, burns or frostbite. Falls are the leading cause of late (i.e., 12 or more weeks after adverse event) amputations due to infections, non-union fractures, and painful deformations [10]. Causes of amputations change over time as demonstrated in Cambodia where before 2000, war-related causes were the leading reason for limb loss, after that, non-communicable diseases and road accidents [11]. With globally changing disease patterns, causes for limb amputations are likely to change, with service needs increasing globally, including travel and travel health.

### Prosthetics

Prosthetics is a branch of medicine, specifically surgery, concerned with replacing missing or lost body parts. The realm of prostheses provides a range of options to users—based on their level of limb difference—varying in cosmesis and functionality to cater to their unique needs and lifestyles [12]. Replacing a missing limb with an artificial gadget (prosthesis) goes back to antiquity. Historically, the key motivations have been cosmesis, occupation, and/or personal self-sufficiency. The oldest known prostheses are Egyptian replacements of big toes made of wood and leather, which are not only imperative for restoring the spiritual requirement for bodily wholeness in life and the afterlife; a big toe was indispensable for wearing local sandals. From ancient Greece and Rome onwards, crude artificial limbs made of wood and metal enabled those injured in battle to take up arms again. Basic hook hands and peg legs (crutches with wood or leather cups), popularised in pirate movies, remain a design feature until today though with highly advanced materials. Artificial limbs, e.g., ‘iron hands’, were worn not only for cosmetic reasons but, with some pulleys and joints, for rudimentary function. Barber-surgeon Ambroise Paré (1510-1590CE) is credited with a completely new approach to wound management, especially gunshot wounds, prevention of haemorrhage during amputations and the design of artificial limbs with acceptable functionality to replicate human movement [13–15]. The following centuries saw constant refinement in appearance, materials, functionality, power source, asepsis, and better surgical procedures, further spurred by the two World Wars [15].

Today’s modern, clinically viable prostheses consist of an external socket to interface with the body, a suspension/attachment system (e.g., suction, harness), relevant joints, an outer cover, and the terminal functional attachment (hand, hook, foot, activity-specific device, e.g., for running, climbing, sport). Prostheses may be passive (e.g., lifelike replacements of a limb only) or active, e.g., body-powered (with harness and cable), externally powered (battery-powered motor where microprocessors relay instructions generated by body movements or through myoelectric signals), or robotic. The needs and requirements of UL and LL prostheses users are varied.

Various prosthesis types have witnessed different levels of maturity and refinement [16] and are prone to varying levels of repair and maintenance requirements. UL prostheses, while rarer, require much more sophistication to replace intricate hand dexterity and arm movements while considering dominant vs. non-dominant limb loss. The type of prosthesis depends on proximal vs. distal levels of amputation, e.g., for UL, any level between partial finger(s) to shoulder disarticulation. Technological advances continue to make simple and intuitive devices [17–19].

### Psychological issues and body image

People with four intact limbs will probably never fully understand the impact of losing part of one’s body. The psychological factors of limb loss and prosthesis acceptance are enormous and well-discussed in the literature. Body image is part of our identity. Prostheses can have a considerable psychological and physical impact on a person across many aspects of their life, provide a sense of bodily completeness and support gendered identities [20]. Further, prosthetic devices are vital for users as they aid them in appearing and living life ‘like everybody does’ and being treated ‘like everyone else’, allowing independent travel and helping with participation in social activities [21]. Adjusting to a major alteration is complicated and influenced by the reasons for amputation, e.g., a sudden loss out of one’s control due to trauma, a considered decision after years of failing other treatments, or a necessary life-saving choice. Several factors influence adaptations [22]. Amputation-specific factors include ‘disfigurement’, altered body movement or loss of self-presentation, e.g., the inability to wear high heels or certain types of clothing. In contrast, a prosthetic for congenital limb loss alters the ‘normal’ (limb-less) body the opposite way. Children and older adults appear to accept prostheses better than, for example, adolescents with concerns about cosmetics and social acceptance; others cope with ‘wear it loud and proud’ [22] expressed in fashionable colourful prostheses. UL amputations appear the most challenging since arms and hands are crucial for essential activities, such as personal hygiene, food preparation, non-verbal communication, and innumerable others. UL prostheses are highly noticeable and less functional than LL prostheses, as achieving satisfactory control has been challenging. Most UL amputations result from trauma, and more than 70% of amputees have problems due to appearance, weight, sweating and functionality [22]. Despite technological advances in UL prostheses, discomfort and weight led to up to 50% of prosthesis abandonment [23].

A phenomenological study of patients four months after amputation describes the psychological impact of the life-changing event [24]. Six themes emerged from the conversations: (1) emotional impact (despair, wish for death), (2) negative emotions (anxiety, lack of control, sadness, anger, unfair fate), (3) tendency towards isolation (social withdrawal, depression, guilt relating to previous life-style choices), (4) role constraints and limitations (struggle with resuming previous roles, including travel, losing family and social ties), (5) phantom limb (including danger of falling due to false sensations), and (6) emotional balancing (tackling new life with determination and hope, e.g., adapted car, resuming previous activities, such as sports and hobbies).

### Rehabilitation and outcome

Two recent studies explored patients’ concerns relating to LL prosthetic rehabilitation. One focused on patients’ expectations of meaningful rehabilitation outcomes [25]. An important expectation was the ability to complete everyday activities like any other person in the way they wanted to, i.e., being independent, not falling over and getting by with as few aids as possible. Other expectations included good pain management and the ability to accept necessary changes, e.g., getting to the toilet at night without prostheses (’scooting on a cushion to the toilet’, p.8). Fundamental, however, is a comfortable and easy-to-use prosthesis, the focus of the second study [26]. Fitting a new prosthesis can be frustrating due to changes in limb shape and volume. The traditional (monocoque) socket is rigid, causing pain and discomfort, potentially leading to pressure sores or friction burns. In addition to the surgical scar, soft tissue, bony prominence, excess skin, and even open wounds can lead to a change in the body’s biomechanics, musculoskeletal overuse-injuries and falls. The weight of prostheses is a problem as is finding suitable liners and socks, with silicon liners (for suction suspension) prone to causing allergic reactions. A major inconvenience is sweat, pooling in sockets, rising over the socket top and staining clothes. To overcome these issues, adjustable sockets have been increasingly fitted to patients in the recent past to allow them to customise fit based on residuum volume fluctuation to improve their comfort and gait stability. Travel could be uncomfortable, hence avoided, due to lack of accessibility, uneven surfaces, potential falls with nobody around to help, and difficulty managing pain, heat, odour, and sweat (‘…can’t … [in public] …taking your leg off and wiping it all and everything…’, p.65). Elsewhere, 2–3 months after rehabilitation discharge, the greatest decrease in participation occurred in outdoor and travel activities [27].

### Self-management

To avoid secondary complications and improve QOL, long-term self-management of living with a prosthesis includes caring for residuum, the prosthesis, and their interface. There is a lack of high-quality research into the most appropriate interventions currently suggested in textbooks, patient handouts and by relevant organisations [28]. Skin problems, e.g., mechanically induced, allergic reactions or bacterial/fungal infections [29] and hygiene issues, e.g., skin eruptions, sweating, itching, and odour [30] are recurring concerns in need of much more investigations. A large range of detailed practical advice seems to stem generally from amputees or health professionals to be tailored to everyone as needed, e.g., caring for the intact and residual limb [31, 32], caring for UL and LL prosthetics [33, 34], and how to avoid falls [35].

This paper aimed to review the academic literature for preferably evidence-based travel-related guidelines with practical advice for people with prosthetic limbs. Such evidence enables travel health professionals to appreciate a traveller’s complex circumstances and to incorporate such insight into high-quality travel health consultations.

## Method

Although amputees may use wheelchairs as additional mobility aids, travelling with wheelchairs, a large topic in itself, is excluded here, as are orthoses and osseointegrated prostheses. All major databases were searched by a specialist librarian and later again by the authors, using various keyword combinations of or actual: (travel OR touris*) AND (“artificial arm” OR “artificial arms” OR “artificial extremities” OR “artificial extremity” OR “artificial leg” OR “artificial legs” OR “artificial limb” OR “artificial limbs” OR prosthetic* OR “robotic arm” OR “robotic arms” OR “robotic extremities” OR “robotic extremity” OR “robotic leg” OR “robotic legs” OR “robotic limb” OR “robotic limbs” OR prostheses OR prosthesis), without language or date restrictions. It turned out - the term ‘travel’ in this context stands for ambulation with a lower limb prosthesis, i.e., a certain distance across a room or on a treadmill, not travel in the sense of going somewhere. The search yielded zero results. Reference lists of articles on amputation rehabilitation were searched unsuccessfully for any paper of interest. Relevant journals in fields such as travel medicine, tourism, and disability/prosthetics were searched individually without results. Selected authors and university departments were approached directly via snowballing, yielding valuable background information but little on travelling with prosthetic limbs.

The unsuccessful search for academic papers directed the focus to grey literature, especially publications by relevant organisations, professional bodies, online contributions by amputees and/or caregivers, manufacturers, self-help groups, government websites, airlines, the Australian Paralympian Committee, prosthetic and physiotherapy clinics, as well as incidental communications with amputees, including veterans and athletes. Contacting all country members of an international association had limited success. All information was collected until data saturation and categorised into useful sections providing sufficient content for preliminary guidelines for travel health practitioners and a baseline for urgently needed research.

## Results

### Travel health advice for travellers with limb prostheses

People may travel to a neighbouring country to visit family, fly to the other end of the world for a luxury retreat, or from one village to the next market town. They may come from low-income regions to a place with modern amenities, e.g., the annual Hajj, or from a wealthy place staying in rustic huts along a remote mountain trail. If they are amputees, they will share common challenges, the difference being not just their personal history but the availability of funds, from the type of prosthesis and preparative service at home to the level and quality of transport and accommodation, maintenance, and insurance issues during a trip. Due to space constraints, this paper will focus on travel by plane, even though other transport modes, such as ship, car, bus, or train, are equally important, e.g., large train stations with long platforms, high steps on carriages and so on. Much that is discussed here is easily transferable to other situations.

A traveller with a prosthesis consulting a travel clinic may already have gone through much suffering [36]. The background information in the introduction lets travel health professionals acknowledge and apply appropriate sections to their travel care. Pre-travel care for children with paediatric prostheses [9] will be guided by the accompanying parents’ experiences. Travellers receive the usual general and destination-specific travel health care, such as vaccinations (potentially re-considering the injection site), while recognising additional aspects of wearing prosthetic limbs. Most travel hints and tips suggested below are either general or refer to LL prostheses and are often based on desperation and trial-and-error or are of the ‘always done it that way’ kind without evidence-base. Problems arise around falls, walking on challenging surfaces (e.g., cobblestones, sand dunes, ancient ruins, or slippery ice), steps, (shared) bathrooms, managing heat and humidity (reduced body surface due to a missing limb affects temperature regulation), clothing issues in frigid weather, carrying loads (luggage) that exceed the prosthesis’s capacity, and skin care and hygiene regimens. Adding to the challenge is the higher energy expenditure of LL amputees compared to non-disabled individuals [37] to walk and keep balance and posture [38]. UL amputees have a particularly tough time travelling not only if their dominant limb is missing but also coping with a myriad of actions required, such as personal hygiene, eating/drinking, (squat) toilets, standing in full buses, holding ticket and boarding pass, carrying luggage or getting up from awkward seats. The suggested hints and tips for travellers with prostheses are divided into the following four sections.

### Preparation

Travellers need to go through the usual steps of travel preparation with particular emphasis on planned activities, prostheses, and insurance. By far the most crucial aspect is the preparation relating to accessibility, availability and troubleshooting at the destination (Table 1).

**Table 1:** Suggested Tips for Preparation

**General Preparation**
- General Travel health advice - Check that the prosthesis is in good working order, with no cracks or leaks. - Practice anticipated activity with prosthetic beforehand. - LL: practice getting up after a fall. - Consider foregoing activities that may harm the prosthesis (e.g., water-resistant prosthesis not always suitable for pressure or saltwater in scuba diving). - Prepare some useful phrases in the languages of the destination regarding prosthetics. - Travel insurance covering prosthetics, including overseas repairs. - Take details of a professional at home for remote/telehealth advice. - Are there suitable rental car insurance and equipment?
**Preparation Relating to the Destination**
- Assess the local situation and requirements for the chosen activity, e.g., adventure, climbing, skiing, cycling, theme parks, and others. - Check destination for o prosthetist, o franchises of suppliers/manufacturers and if services be availed seamlessly, if necessary, o repair facilities (active and passive devices require different levels of repair and maintenance), o accessibility of transport and accommodation (lift!), o medical facilities, o availability of reliable power supply for externally-powered prostheses, o voltages for medical equipment, o where to charge the electronic prosthesis. Pack power box (or use mechanical prosthesis), o weather (humidity/heat) and travel plans (prosthesis may be more challenged than in everyday life) - Are guides/organisers aware of the requirements of travellers? - Consider stigma/cultural differences/discrimination surrounding disability (important for first-time travellers). - In Europe, obtain EuroKey [39] to open toilets. - Public swimming pools: where to leave a very expensive prosthesis? - Avoid sand-based activities with myoelectric prosthesis.

### What to pack?

Apart from personal belongings, much luggage space is dedicated to prostheses, skin care and additional items (Table 2). Weight limits may prove challenging. In the US, carry-on bags with essential medical devices/spare parts ONLY do not attract extra charges.

**Table 2:** Suggested Tips for Packing

**Prosthesis**
- Prosthetic suitable for tourism activity, e.g., especially robust construction. - Activity-specific devices, e.g., hand or feet for purpose (hiking, running, surfing, climbing). - Spare parts, tools, batteries, chargers, and suction valve (consider possible issues with sharp/metal items in a carry-on bag to avoid repacking and restrictions regarding batteries). - Spare prosthesis/water-resistant prosthesis (carry-on). - Liners, water-resistant liners, covers (swimming, surfing), stump socks, stump shrinkers, compression socks, and plastic covers for electric parts in wet weather. - Suspension sleeves (to hold prosthesis onto leg).
**Skin care**
- Prosthetic soap (mild antibacterial) to keep skin fresh. - Baby powder (hot/humid) to keep skin dry. - Disinfectant wipes, rubbing alcohol, skin ointment/lotion, and antibacterial creme. - Sunblock for stump (usually unaccustomed to the sun). - Hand mirror for regular stump inspection.
**Other**
- Cleaning supplies (esp. for prosthetic skin as stains are hard to remove). - Duct tape. - Consider the addition of mobility aids, e.g., travel crutches, foldable walking sticks, wheelchair. - If using flip-flops, double-sided adhesive tape to the heel keeps the shoe in place. - For beach holidays: small device/hairdryer to blow sand out of joints. - Special well-cushioned prosthesis bag if checked in luggage.

### Travel by plane

Unfortunately, there have been numerous instances of people with disabilities facing dehumanising experiences while navigating airports or flights [40]. The Australian Government Civil Aviation Safety Authority’s website offers advice for flying with a disability but nothing on prostheses [41–43]. Most airlines seem equally quiet on this matter, offering small amounts of information on wheelchairs. Several aspects contribute to a pleasant flying experience [44]. An important factor for travellers with impaired mobility is the (dis)comfort of plane seats, especially in economy class. Such problems, e.g., pressure sores, have been studied with Paralympic athletes who use wheelchairs [45], but there seems to be little work done for prosthesis-wearers. Studying seat comfort for ‘able’ passengers, the seat pitch, i.e., the distance from one point on a seat to the same point on a seat a row in front or behind, which influences the all-important legroom, appeared highly important [46]. The optimal seat pitch is 86–106 cm (34–42 inches) to allow stretching and changing position, thereby also lowering the risk of deep vein thrombosis (DVT). Beyond that distance, discomfort increases again [47]. Therefore, passengers with prostheses will be especially interested in comfortable seats and choose airlines accordingly, if possible, to avoid secondary complications on long-haul flights. Furthermore, the ability to use narrow toilet facilities (with non-standard fittings), typically designed for non-disabled/intact-limbed individuals needs consideration. Airlines should also seek input from prosthetic limb users, occupational therapists, and prosthetic and orthotic (P&O) professionals when it comes to meal, drinks or cutlery preferences for UL prosthesis users as eating/drinking certain items or those requiring bimanual ability might be challenging. Navigating airports poses additional challenges, though some airports may offer assistance, e.g., Brisbane Airport’s ‘Hidden Disabilities’ program handing out sunflower lanyards for personnel to recognise passengers with potential support needs, especially when travelling alone.

Some tips are general (usual residuum [commonly known as stump] care) or specific for LL amputees (compression garment, also to avoid blisters as stumps swell due to cabin pressure and inactivity or opting for a prosthesis with an adjustable socket) (Table 3a). Additional considerations for UL amputees are presented in Table 3b.

**Table 3a:** Suggestions for Travelling by Plane (general and lower limb)

**Airport**
- Consider long distances in airports, stairs, and trains. - Consider long waiting queues. - Pre-book assistance. - Ask for early boarding at the gate. - Book a wheelchair or buggy to get to the gate quickly/early. - Choose a flight connection with ample time for lay-over.
**Airline**
- Store limbs in an overhead locker without alarming passengers. - Airline restrictions: Air New Zealand: only one limb carry-on; some airlines do not allow any. - Airlines are supposed to assist with boarding, connection to other flights, seating, storing carry-ons, and handling service-animals.
**Seat**
- Seat closer to toilet/aisle. - Book bulkhead seating or seating with extra legroom to move (DVT), prosthetic side facing the aisle for ease of ingress/egress as preferred. - Sitting in the exit row is usually prohibited.

**Table 3b:** Suggested Tips for Travelling by Plane (additions for upper limb)

- Handle luggage, carry-on, passport, boarding card, wallet
- Be ready for security (e.g., take off jacket/shoes, take out laptop/devices) - Choose a seat with the prosthetic away from the aisle so meals and drinks can be received more easily. Others may prefer the prosthetic near the aisle for more space. - Check if the armrest can be lowered/removed. - The armrest may be needed for support to get up. - Choice of a window seat on the side of the residual limb or the prosthetic side as per preference (space, comfort). - Carry-on bag vs. backpack? - For powered bionic limbs: o The Bluetooth connection must be switched off during flights whenever mobile phones must be switched off or in flight mode [48]. o Proximity to sources of strong magnetic or electrical interference, such as theft prevention systems (e.g., airport shops) or metal detectors (e.g., airport security) may cause electromagnetic interference, potentially leading to injury due to unexpected behaviour of the prosthesis system [49].

### Airport security

A large amount of advice centres around getting through airport security, yet this part of travel also receives bitter complaints. Governments regulate airport security procedures and issue protocols for screening passengers with limb prostheses. In many high-income countries, staff are trained to follow strict guidelines relating to the screening process. Travellers must not be asked to remove their prostheses, artificial limbs must not be touched, and shoes should not have to be removed, for the risk of falling or slipping. Further investigation, if warranted, must happen in a private room. Unfortunately, security staff does not always follow the prescribed guidelines. Amputees’ complaints included the humiliating removal of prostheses in front of other passengers or having to crawl through the metal detector. Travellers should expect ‘trouble’ and ask to see the supervisor, who might be better informed.

Travellers should arrive at the airport in plenty of time, consider items that may need removing, e.g., emptying pockets, removing jackets or belts, and wear comfortable clothing, perhaps zip-off cargo pants or trousers with zips along the inner length, allowing for a quick inspection. Preparations and screening will be more testing for travellers with more than one limb deficiency. Travellers should advise security personnel of the prosthesis before going through the screen. In the US, the Transport Security Administration (TSA) offers notification cards and pre-checks to simplify the procedure. However, travellers should be prepared that their foreign destination airport may have no specific guidelines or staff not adhering to them or being trained.

## Discussion

To our knowledge, this is the first paper on travel with a prosthetic limb in any field, including travel medicine. There is now a reasonable body of knowledge on travel with a disability in general or focused on wheelchair users, and many tour operators cater for them. The dearth of literature catering to travelling with a UL/LL prosthesis might explain why travel medicine has so far neglected prosthetic limb users. Sports organisations and special group travel operators may have a team doctor or prosthetist. Individual travellers, especially first-time travellers, with a prosthesis need to fend for themselves or rely on other amputees’ experience, self-help groups or organisations’ factsheets, and use a trial-and-error approach, which could sully the travel experience. Travel medicine has missed an entire special group of travellers but is in an excellent position to remedy this oversight. Several recommendations arise from this paper for practice, education, and research, not only for travel health clinicians but also for device manufacturers, P&O service providers, healthcare insurers, travel insurers, and others.

### Recommendations for clinical practice

Travel health practitioners should obtain from the traveller a full picture of the travel plans and their daily life with a prosthesis, including practical difficulties and their coping strategies. Travel health advice must acknowledge the circumstances of a traveller with limb prosthesis including, at times, the sensible advice to not proceed with or alter travel plans as is sometimes necessary for other health conditions. Travel-related suggestions could be made in conjunction with the traveller’s prosthetist or (multidisciplinary) rehabilitation teams. For certain amputations, injection sites for vaccines may need to be altered. Updated medical and technical information should be sought by connecting with prosthetists, physiotherapists, psychologists, and other relevant health professionals.

### Recommendations for education

The special groups of travellers with prosthetic limbs should be included in travel medicine short and degree courses, initially to raise awareness and convey content as is currently available, progressively replacing information with evidence-based knowledge. Clinic staff also should receive relevant training. As P&O education continues to evolve with a focus on patient/client-centred care [50], adding travel-related content, ideally in cooperation with travel health clinicians, would promise benefits for the two specialist fields and, most importantly, for travellers with artificial limbs.

### Recommendations for research

We found no peer-reviewed literature on travel health advice for prosthetic limb users. In a field with a limited body of knowledge, an inductive approach is recommended. Qualitative studies to understand the lived experience of people with limb prostheses form the basis for further investigations. We do not yet understand the travel health knowledge, attitudes and practices among travellers using prosthetic limbs, warranting dedicated research. How limb difference affects travel plans (domestic/international, group/solo) should incorporate all age groups, genders, socio-economic status and amputation levels, including unilateral/bilateral and dominant/non-dominant limb loss. What kind of travel health advice amputees expect is unknown, as is their experience with the advice received in the past. Practical issues arising from the hints and tips mentioned under ‘Results’ open a wide range of potential research topics amenable to a range of exciting research methods. On location, barriers, problems, and concerns could be collected via visual data collection methods. Challenges arising from extreme tourist activities, e.g., high altitude, scuba diving, or long-distance hiking, are unknown. More knowledge is needed on using additional assistive devices by travellers with prostheses, e.g., wheelchairs, crutches, and other walking aids. Other prostheses, such as ocular, internal joint or breast prostheses, rate no mention at all concerning travel. It will also be helpful to develop a dedicated travel medicine mobile phone app for prosthesis users in the future. On a different note, it would be interesting to understand the extent of medical tourism in prosthetic rehabilitation.

### Limitations

What was originally intended to be a literature review on travel with prosthetic limbs turned into a ‘scooping’ rather than ‘scoping’ exercise due to an utter lack of high-quality peer-reviewed work. Apart from one contribution in German, only English-language material was available. Studies published in other languages possibly exist, especially in countries with conflict-related amputations, but they will have been missed. Such authors should be encouraged to share their work more widely. Nevertheless, this paper offers plenty to work with.

## Conclusion

Travellers with prosthetic limbs seem off the radar in relevant disciplines, including travel medicine. This paper provided some background information enabling travel health practitioners to appreciate amputees’ unique circumstances and how those may influence travel and tourism activities. A collection of currently suggested pieces of advice may inform individually tailored travel health advice. More importantly, these suggestions offer a wide range of potential research topics for urgently needed evidence-based guidelines for a group of travellers who have, so far, missed out on the attention of travel medicine.

## Data Availability

No datasets were generated or analysed during the current study.

## Abbreviations

CISTM: Conference of the International Society of Travel Medicine
DVT: Deep vein thrombosis
LL: Lower limb
P & O: Prosthetic and orthotic
QOL: Quality of life
TSA: Transport Security Administration
UL: Upper limb
WHO: World Health Organisation

## References

[1] Chen C, Petrick J. Health and wellness benefits of travel experiences: a literature review. J Travel Res. 2013;52:709–19. 10.1177/0047287513496477. 10.1177/0047287513496477

[2] Bauer I. When travel is a challenge: travel medicine and the ‘dis-abled’ traveller. Travel Med Infect Dis. 2018;22:66–72. 10.1016/j.tmaid.2018.02.001. 29454050 10.1016/j.tmaid.2018.02.001

[3] Moura A, Eusébio C, Devile E. 2023. The ‘why’ and ‘what for’ of participation in tourism activities: Travel motivations of people with disabilities. Curr Iss Tour 2023; 26:941 – 57, 10.1080/13683500.2022.2044292.

[4] Poria Y, Reichel A, Brandt Y. The flight experiences of people with disabilities: an exploratory study. J Travel Res 2010; 49:216 – 27, doi: 0.1177/0047287509336477.

[5] De Pascale A, Meleddu M, Abbate T, Pellicano M. Is there a gender gap in the propensity to travel of people with disabilities? J Travel Res. 2023;62:517–39. 10.1177/00472875211073976. 10.1177/00472875211073976

[6] Jander C, Martin L. Guidelines for travelling with passengers with a disability. J Aust Soc Aerosp Med. 2015;10:22–5. 10.21307/asam-2015-008. 10.21307/asam-2015-008

[7] Urner C. Passenger travel on freighters. In: Steffen R, Lobel H, Haworth J, Bradley D, editors. Travel medicine: proceedings of the first conference on international travel medicine, Zürich, Switzerland, 5–8 April 1988. Berlin: Springer; 1989, 468 – 73.

[8] Cieza A, Causey K, Kamenov K, et al. Global estimates of the need for rehabilitation based on the Global Burden of Disease study 2019: a systematic analysis for the global burden of Disease Study 2019. Lancet. 2021;396:2006–17. 10.1016/S0140-6736(20)32340-0. 33275908 10.1016/S0140-6736(20)32340-0 PMC7811204

[9] Ziegler-Graham K, MacKenzie E, Ephraim P. Estimating the prevalence of limb loss in the United States: 2005 to 2050. Arch Phys Med Rehabil. 2008;89:422–9. 10.1016/j.apmr.2007.11.005. 18295618 10.1016/j.apmr.2007.11.005

[10] McDonald C, Westcott-McCoy, Kartin D, et al. Global prevalence of traumatic non-fatal limb amputation. Prosthet Orthot Int. 2020;0:1–12. 10.1177/0309364620972258. 10.1177/0309364620972258 33274665

[11] Dickinson A, Gates L, Metcalf C, et al. Learning about the changing needs for prosthetics service provision from routinely collected digital centre management data: an exemplar study across three clinics in Cambodia. J Global Health. 2022;12:04083. 10.7189/jogh.12.04083. 10.7189/jogh.12.04083 PMC9579830 36259231

[12] Kyberd P. Making hands: a history of prosthetic arms. Academic; 2021. 10.1016/C2019-0-01285-X.

[13] Finch J. The ancient origins of prosthetic medicine. Lancet. 2011;377:548–9. 10.1016/S0140-6736(11)60190-6. 21341402 10.1016/S0140-6736(11)60190-6

[14] Thurston A. Paré and prosthetics: the early history of artificial limbs. ANZ J Surg. 2007;77:1114–9. 10.1111/j.1445-2197.2007.04330.x. 17973673 10.1111/j.1445-2197.2007.04330.x

[15] Zuo K, Olson J. The evolution of functional hand replacement: from iron prostheses to hand transplantation. Plast Surg. 2014;22:44–51. 10.1177/229255031402200111 PMC4128433 25152647

[16] Maat B, Smit G, Plettenburg D, Breedveld P. 2017. Passive prosthetic hands and tools: A literature review. Prosthet Orthot Int 2017; 42:66–74, 10.1177/0309364617691622. 10.1177/0309364617691622 PMC5810914 28190380

[17] Nagaraja V, da Ponte Lopes J, Bergman J. Reimagining prosthetic control: a novel body-powered prosthetic system for simultaneous control and actuation. Prosthesis. 2022;4:394–413. 10.3390/prosthesis4030032. 10.3390/prosthesis4030032

[18] Nagaraja V, Moulic S, D’Souza J, et al. A novel respiratory control and actuation system for upper-limb prosthesis users: clinical evaluation study. IEEE Access. 2022;10:128764–78. 10.1109/ACCESS.2022.3226697. 10.1109/ACCESS.2022.3226697

[19] Hashim N, Abd Razak N, Osman N, Gholizadeh H. Improvement on upper limb body-powered prostheses (1921–2016): a systematic review. Proc Inst Mech Eng Part H: J Eng Med. 2017;232:3–11. 10.1177/095441191774458. 10.1177/095441191774458 29199518

[20] Murray C. Embodiment and prosthetics. In: Gallagher P, Desmond D, MacLachlan M, editors. Psychoprosthetics. London: Springer; 2008. pp. 119–29. 10.1007/978-1-84628-980-4_9.

[21] Murray C. Being like everybody else: the personal meanings of being a prosthesis user. Disabil Rehabil. 2009;31:573–81. 10.1080/09638280802240290. 19034778 10.1080/09638280802240290

[22] Rybarczyk B, Behel J. Limb loss and body image. In: Gallagher P, Desmond D, MacLachlan M, editors. Psychoprosthesis. London: Springer; 2008. pp. 23–31. 10.1007/978-1-84628-980-4_3.

[23] Salminger S, Stino H, Pichler L, et al. Current rates of prosthetic usage in upper-limb amputees - have innovations had an impact on device acceptance? Disabil Rehabil. 2022;44:3708–13. 10.1080/09638288.2020.1866684. 33377803 10.1080/09638288.2020.1866684

[24] Roşca A, Baciu C, Burtăverde V, Mateizer A. Psychological consequences in patients with amputation of a limb. An interpretative-phenomenological analysis. Front Psychol. 2021;12:537493. 10.3389/fpsyg.2021.537493. 34122200 10.3389/fpsyg.2021.537493 PMC8189153

[25] Ostler C, Donovan-Hall M, Dickinson A, Metcalf C. Exploring meaningful outcome domains of recovery following lower limb amputation and prosthetic rehabilitation: the patient’s perspective. Disabil Rehabil. 2022;1–14. 10.1080/09638288.2022.2138989. 10.1080/09638288.2022.2138989 36368639

[26] Turner S, Belsi A, McGregor A. Issues faced by people with amputation(s) during lower limb prosthetic rehabilitation: a thematic analysis. Prosthet Orthot Int. 2022;46:61–7. 10.1097/PXR.0000000000000070. 34789709 10.1097/PXR.0000000000000070 PMC8865619

[27] Couture M, Caron C, Desrosiers J. Leisure activities following a lower limb amputation. Disabil Rehabil. 2010;32:57–64. 10.3109/09638280902998797. 19925277 10.3109/09638280902998797

[28] Lee DJ, Repole T, Taussig E, et al. Self-management in persons with limb loss: a systematic review. Can Prosthet Orthot J. 2021;4:5. 10.33137/cpoj.v4i1.35098. 10.33137/cpoj.v4i1.35098 PMC10443519 37614928

[29] Meulenbelt H, Geertzen J, Jonkman M, Dijkstra P. Skin problems of the stump in lower limb amputees: 1. A clinical study. Acta Derm Venereol. 2011;91:173–7. 10.2340/00015555-1040. 21290085 10.2340/00015555-1040

[30] Hachisuka K, Nakamura T, Ohmine S, et al. Hygiene problems of residual limb and silicone liners in transtibial amputees wearing the total surface bearing socket. Arch Phys Med Rehabil. 2001;82(9):1286–90. 10.1053/apmr.2001.25154. 11552206 10.1053/apmr.2001.25154

[31] New South Wales Government. Health Share EnableNSW – Prosthetic Limb Service. October 2015. Caring for your residual and intact limb. https://www.enable.health.nsw.gov.au/about/publications/fact-sheets/caring-for-your-residual-and-intact-limb (accessed 20.7.2023).

[32] Limbs4Life. Caring for your residual limb. https://www.limbs4life.org.au/prosthetics/funding-for-prosthetic (accessed 20.7.2023).

[33] New South Wales Government. Health Share EnableNSW – Prosthetic Limb Service. Caring for your prosthetic upper limb. https://www.enable.health.nsw.gov.au/about/publications/fact-sheets/caring-for-your-prosthetic-upper-limb (accessed 20.7.2023).

[34] New South Wales Government. Health Share EnableNSW – Prosthetic Limb Service. Caring for your prosthetic lower limb. https://www.enable.health.nsw.gov.au/about/publications/fact-sheets/caring-for-your-prosthetic-lower-limb (accessed 20.7.2023).

[35] New South Wales Government. Health Share EnableNSW – Prosthetic Limb Service. October 2015. How to avoid a fall. https://www.enable.health.nsw.gov.au/about/publications/fact-sheets/how-to-avoid-a-fall (accessed 20.7.2013).

[36] Dillon M, Anderson S, Duke E. The lived experience of sequential partial foot and transtibial amputation. Disabil Rehab. 2020;42:2106–14. 10.1080/09638288.2018.1555288. 10.1080/09638288.2018.1555288 30669884

[37] Schmalz T, Blumentritt S, Jarasch R. Energy expenditure and biomechanical characteristics of lower limb amputee gait: the influence of prosthetic alignment and different prosthetic components. Gait Posture. 2002;16:255–63. 10.1016/s0966-6362(02)00008-5. 12443950 10.1016/s0966-6362(02)00008-5

[38] Hoffman M, Sheldahl L, Buley K, Sandford P. Physiological comparison of walking among bilateral above-knee amputee and able-bodied subjects, and a model to account for the differences in metabolic cost. Arch Phys Med Rehabil. 1997;78:385–92. 10.1016/s0003-9993(97)90230-6. 9111458 10.1016/s0003-9993(97)90230-6

[39] EuroKey. https://www.eurokey.ch/en/index.html (accessed 17.7.2023).

[40] Swartz L, MacLachlan M. From the local to the global: the many contexts of disability and international development. In: MacLachlan M, Swartz L, editors. Disability & international development: towards inclusive global health. New York: Springer; 2009. pp. 1–11.

[41] Australian Government Civil Aviation Safety Authority. Planning for travel with a disability. 13.7. 2022. https://www.casa.gov.au/operations-safety-and-travel/travel-and-passengers/passengers-disability-and-reduced-mobility/planning-travel-disability (accessed 15.6.2023).

[42] Australian Government Civil Aviation Safety Authority. Wheelchairs and mobility aids. https://www.casa.gov.au/operations-safety-and-travel/travel-and-passengers/passengers-disability-and-reduced-mobility/wheelchairs-and-mobility-aids (accessed 15.6.2023).

[43] Australian Government Civil Aviation Safety Authority. Boarding your flight. https://www.casa.gov.au/operations-safety-and-travel/travel-and-passengers/passengers-disability-and-reduced-mobility/boarding-your-flight (accessed 15.6.2023).

[44] Vink P, Bazley C, Kamp I, Blok M. Possibilities to improve the aircraft interior comfort experience. Appl Ergon. 2012;34:354–9. 10.1016/j.apergo.2011.06.011. 10.1016/j.apergo.2011.06.011 21803331

[45] Hayden D, Wilson S, Faulkner K. Long haul travel seating: impact of seating interfaces. Sydney: Australian Paralympic Committee; 2016.

[46] Anjani S, Li W, Ruiteer I, Vink P. The effect of aircraft seat pitch on comfort. Appl Ergon. 2020;88:103132. 10.1016/j.apergo.2020.103132. 32678792 10.1016/j.apergo.2020.103132

[47] Kremser F, Guenzkofer F, Sedlmeier C, et al. Aircraft seating comfort: the influence of seat pitch on passengers’ well-being. Work. 2012;41(Suppl 1):4936–42. 10.3233/WOR-2012-0789-4936. 22317483 10.3233/WOR-2012-0789-4936

[48] Open Bionics. Hero arm user Manual Version 12.0. Open Bionics: Bristol; 2023.

[49] Axon-Bus Prosthetic System. Manual. Otto Bock: Vienna; 2021.

[50] Spaulding S, Kheng S, Kapp S, Harte C. Education in prosthetic and orthotic training: looking back 50 years and moving forward. Prosthet Orthot Int. 2020;44:416–26. 10.1177/0309364620968644. 33164659 10.1177/0309364620968644

